# The association between digital communication tool use and perceived mental health among older adults in Canada

**DOI:** 10.1371/journal.pgph.0006540

**Published:** 2026-06-24

**Authors:** Safoura Zangiabadi, Durmalouk Kesibi, Hossam Ali-Hassan

**Affiliations:** 1 School of Kinesiology and Health Sciences, York University, Toronto, Ontario, Canada; 2 Department of Economics, Business, and Math, Glendon campus, York University, Toronto, Ontario, Canada; McGill University, CANADA

## Abstract

This study examines the association between digital communication tool use and perceived mental health among older adults aged 55 years and older in Canada, using data from the 2022 Canadian Internet Use Survey. The sample included 13,536 adults, weighted to represent 12,735,479 individuals. Perceived mental health was self-rated and assessed using a 5-point Likert scale. Digital communication tool usage was measured by reported engagement in activities such as sending emails, using instant messaging apps, accessing social networking sites, making online calls, and uploading content. Multiple linear regression models were used to examine the associations between digital communication tool usage and perceived mental health, adjusting for sociodemographic factors. Results indicated that over half of older adults engaged in digital communication. Use of social networking sites was associated with poorer perceived mental health (Adjβ = -0.080; p = 0.002), whereas email use was positively associated with perceived mental health (Adjβ = 0.113; p < 0.001). These findings may inform policies and interventions to support the mental health of older adults in the digital era.

## Introduction

The rapid advancement of the digital technologies has extensively transformed the way individuals communicated and interacted across all age groups. While younger generations have seamlessly integrated digital tools into their daily lives, older adults have also increasingly adopted the use of digital technologies such as the internet, digital communication tools, and social networking sites in the recent years [1]. The use of online social networking sites (e.g., Facebook, Twitter) and technology-based communication tools (e.g., e-mail, instant messaging) enables older adults to stay connected with family and friends, maintain social ties, and access information and care services [1,2]. In 2022, 83% of Canadian seniors used the Internet, engaged in various online activities and were considered as the fastest growing group on the Internet [3]. The accessibility and availability of digital tools offer older adults an alternative means of social engagement and communication, particularly when traditional forms of socialization may be restricted due to mobility constraints, retirement, or geographical distance [4]. Furthermore, Internet-based interventions have shown promising potential in supporting the management of chronic diseases, facilitating lifestyle modifications, and promoting overall health. For instance, during the pandemic there was a widespread adoption of telehealth technologies among older adults to maintain access to essential healthcare services which was found to be beneficial for individuals with limited mobility and helped reduce the risk of virus transmission [5,6]. Subsequently, the use of Internet and digital communication tools has been linked to reduced social isolation and feelings of loneliness, increased social support, and a greater life satisfaction among older adults [7,8].

Despite the potential benefits of the digital communication activities, the literature presents mixed findings on the impact of the digital communication tool us on mental health among older adults. These inconsistencies may be explained by differences in the mechanisms through which digital tools shape social interactions. Specifically, variations in communication timing (real-time versus asynchronous), levels of reciprocity, and patterns of engagement (active versus passive) may differentially influence social support, social comparison, and emotional processing, thereby contributing to differences in mental health outcomes [9,10]. Some studies linked internet use to improved mental health by decreasing social isolation and loneliness. For instance, studies of older adults in China found that internet use reduced depression and feelings of loneliness, while improving cognitive abilities, thereby promoting better mental health in older adults [11–13]. Additionally, a study of older adults in the United States with a mean age of 68 found an association between increased use of online social networking and reduced depressive symptoms, mediated by reduced feelings of loneliness [14]. A review study further suggested that older adults who used the internet for social networking and communication purposes exhibited better mental health during the COVID-19 pandemic [15]. Conversely, some studies suggest that digital behaviors may be associated with poorer mental health outcomes. For example, excessive internet use has been associated with adverse psychological outcomes, including increased symptoms of depression and anxiety, greater social comparison, and reduced face-to-face-interactions, all contributing to poorer overall mental health [11,16]. Additionally, a study of older adults aged 60 and above in the United States found that digital social interactions, such as participating in online communities and discussing health and aging were associated with increased symptoms of anxiety, ultimately impacting their mental health [17]. Given the multifaceted relationship between digital engagement and mental health, further research is essential to explore how various digital communication tools influence the mental health of older adults.

Meanwhile, Canada is experiencing a demographic shift with a rapidly growing aging population. According to Statistics Canada, seniors currently comprise about 19% of the population, or approximately 7.6 million people, and this number is projected to increase to nearly one-quarter of the population by 2030 [18]. Such substantial growth in the number of older adults necessitates adequate measures and resources to meet the care needs of the aging population and to mitigate the financial burden associated with an aging population [19]. Hence, ensuring the mental health and overall well-being of this portion of the population becomes a public health priority. Particularly, mid-to-later adulthood (typically defined as age > 55 years) is characterized by shifts in social roles, networks, and patterns of interaction, influenced by factors such as employment transitions, retirement planning, changes in family structure, and evolving health status. These changes can affect both the form and quality of social engagement. As individuals navigate this life stage, alterations in daily routines and social connections may lead to subtle changes in participation and engagement [20]. Examining this period provides an important opportunity to identify early shifts in social involvement that may have important implications for mental health, before the onset of more pronounced age-related functional limitations. However, existing research on the impact of digital communication tool use on older adults’ mental health remains limited, leaving a gap in knowledge regarding their unique experiences, particularly in the context of Canada’s rapidly aging population.

Understanding how digital communication tools are associated with mental health outcomes can inform policy and practice aimed at supporting mental well-being in this age group. Therefore, this study examines the association between the use of digital communication tools and the self-rated perceived mental health among adults aged 55 years and older in Canada. The findings may inform the development of targeted interventions and strategies to support mental well-being among older adults.

## Methods

This study utilized the data from the 2022 Canadian Internet Use Survey (CIUS) conducted by Statistics Canada between December 2022 and April 2023 [21]. This was a cross-sectional survey aimed at measuring internet access, usage, and the adoption of digital communication tools for online activities among Canadians aged 15 years and older across ten provinces, excluding individuals residing in institutions for more than six months. Participation in the survey was voluntary, with data collected using an electronic questionnaire or computed assisted telephone interviewing (CATI). No identifying information of the participants is included. The overall response rate was 45.3%. For the purposes of this study, the analysis was restricted to individuals 55 and older.

The main outcome of the study was perceived mental health measured by the question “In general, how is your mental health?”. The response options provided ranged from 1 to 5 on a 5-point Likert Scale, with 1 representing “Poor” to 5 representing “Excellent”. The independent variable explored in this study was the use of digital communication tools, measured by the question “In the past three months, which of the following communication-related activities have you conducted online”. Respondents reported on their personal internet use, excluding business and school-related activities in accordance with the survey instructions. Response options included sending and receiving emails, sending messages using instant messaging apps, using social networking websites, making online voice or video calls, using dating websites, and uploading self-created content online. Each response was categorized as either “Yes” or “No”. Additionally, sociodemographic factors such as gender (male/female), age (55–64 years/65 years and over), employment status (yes/no), education (high school or less/some post-secondary/university degree), province (Quebec, Ontario, Manitoba, Saskatchewan, Alberta, British Columbia), immigration status (landed immigrant, non-landed immigrant), number of persons in the household (one person household, two person household, more than two people), and household income (<= $42,256/ $42,257 - $72,366/ $72,367 - $107,480/ $107,481 - $163,750/ > $163,750) were assessed. The information presented on income and immigration status was based on linkage to administrative data sources. Definitions and detailed response categories for all variables are available in the survey questionnaire [21] and a summary can be found in S1 Appendix.

Simple linear regression was performed to examine the bivariate association between use of various digital communication tools and sociodemographic factors with self-rated perceived mental health. Also, multiple linear regression analysis was performed for the outcome of perceived mental health and the main independent variable of digital communication tool use and other sociodemographic variables. The beta coefficient and 95% confidence intervals (95% CI) were reported. Also, statistical significance was set at alpha = 0.05 for all analyses.

To ensure the sample accurately reflects the broader population, all analyses were conducted using population weights applied to each estimate. Bootstrapping techniques were also employed to account for the complex survey design, based on the bootstrap replicate weights supplied with the dataset, further ensuring that the findings are representative at the population level. All statistical analyses were performed using Stata Statistical Software: Release 18 [22]. Ethical review was not required for this study, as the 2022 CIUS public use microdata files are publicly available through Statistics Canada and are protected by legal frameworks such as the Data Liberation Initiative.

## Results

Table 1 summarizes the characteristics of the study participants and the bivariate association between socio-demographic factors and various communication activities with perceived mental health. The analytic sample was restricted to individuals 55 years and older, comprising 13,536 participants, weighted to represent a total population of 12,735,479. Over half (52.15%) were female, and 59% were 65 years or older. The majority, 63.9% were not employed and nearly 60% had had a post-secondary or a university degree. Among participants, (38.22%) resided in Ontario and were non-landed immigrants (83.16%). Additionally, 45.89% of households consisted of two members, and 27.6% reported an annual income of $42,256 or less. More than half reported engaging in communication activities, such as sending and receiving emails (80.21%), using instant messaging applications (59.57%), and accessing social networking websites (52.31%). Regarding perceived mental health, 1.7% of participants rated their mental health as poor, 8.1% as fair, 29.7% as good, 34.7% as very good, and 25.9% as excellent. The overall mean perceived mental health score was 3.75 (SE = 0.01; 95% CI: 3.73, 3.77). The bivariate regression analysis indicated a positive relationship between communication activities including, email communication, instant messaging, and online voice and video calls and perceived mental health (Table 1). Additionally, several sociodemographic factors were positively associated with perceived mental health, including being aged 65 or older, employed, having a post-secondary or university degree, residing in Quebec, being a non-landed immigrant, living in a two-person household, and having an income above $42,257. However, being a female was negatively associated with perceived mental health.

**Table 1:** Weighted characteristics of study participants and the bivariate association between digital communication, and socio-demographic factors with perceived mental health.

Factor	Number	(%)	Perceived Mental Health		
			Unadjusted β (SE)	p-value	95% CI
Activities related to Communication					
Email Communication					
Yes	10,215,652	80.21	0.170 (0.029)	**<0.001**	0.113, 0.227
No	2,468,713	19.38	ref		
Instant Messaging Usage					
Yes	7,586,049	59.57	0.070 (0.022)	**0.002**	0.026, 0.114
No	5,098,316	40.03	ref		
Social Media Engagement					
Yes	6,661,428	52.31	0.012 (0.021)	0.573	-0.030, 0.054
No	6,022,937	47.29	ref		
Online Voice and Video Calls					
Yes	5,653,120	44.39	0.094 (0.021)	**<0.001**	0.052, 0.136
No	7,031,245	55.21	ref		
Use of Dating Platforms					
Yes	443,317	3.48	-0.119 (0.061)	0.053	-0.239, 0.002
No	12,241,048	96.12	ref		
Content Creation and Sharing					
Yes	802,881	6.30	-0.027 (0.047)	0.557	-0.119, 0.064
No	11,881,484	93.29	ref		
Socio-demographic characteristics					
Gender					
Male	6,094,430	47.85	ref		
Female	6,641,049	52.15	-0.052 (0.021)	**0.013**	-0.094, -0.011
Age					
55 to 64 years	5,219,882	40.99	ref		
65 years and over	7,515,598	59.01	0.130 (0.022)	**< 0.001**	0.087, 0.174
Employment status					
Yes	4,131,747	32.44	0.050 (0.022)	**0.023**	0.007, 0.093
No	8,139,406	63.91	ref		
Education					
High school or less	4,721,434	37.07	ref		
Some post-secondary	4,420,534	34.71	0.074 (0.025)	**0.003**	0.025, 0.124
University degree	3,142,925	24.68	0.193 (0.027)	**<0.001**	0.140, 0.245
Province					
Atlantic provinces	971,288	7.63	0.043 (0.029)	0.146	-0.015, 0.101
Quebec	3,064,850	24.07	0.283 (0.028)	**<0.001**	0.227, 0.339
Ontario	4,866,992	38.22	ref		
Manitoba	416,426	3.27	-0.006 (0.048)	0.896	-0.101, 0.088
Saskatchewan	349,596	2.75	-0.049 (0.043)	0.249	-0.133, 0.035
Alberta	1,265,523	9.94	0.004 (0.039)	0.915	-0.073, 0.081
British Columbia	1,800,805	14.14	0.040 (0.033)	0.236	-0.026, 0.105
Immigration status					
Landed immigrant	2,144,747	16.84	ref		
Non-landed immigrant	10,590,732	83.16	0.059 (0.028)	**0.035**	0.004, 0.115
Number of persons in the household					
Single-person household	5,243,537	41.17	ref		
Two-person household	5,843,918	45.89	0.204 (0.022)	**<0.001**	0.160, 0.248
Household of three or more	1,648,024	12.94	0.051 (0.035)	0.143	-0.017, 0.119
Income					
<= $42,256	3,561,148	27.96	ref		
$42,257 - $72,366	3,095,874	24.31	0.195 (0.029)	**<0.001**	0.138, 0.252
$72,367 - $107,480	2,402,653	18.87	0.254 (0.032)	**<0.001**	0.191, 0.317
$107.481 - $163,750	1,937,269	15.21	0.255 (0.034)	**<0.001**	0.188, 0.321
>$163,750	1,738,536	13.65	0.295 (0.035)	**<0.001**	0.226, 0.364

* Sample size is estimated using population weights

#Percentages may not sum to 100% due to missing data

Note: Number = weighted population estimate; % = weighted percentage; unadjusted β = coefficient from survey-weighted bivariate linear regression; SE = bootstrap standard error; CI = confidence interval. All estimates account for the survey design using the main sampling weight and bootstrap replicate weights.

Table 2 presents the findings of the multiple linear regression analysis. All assumptions of multiple linear regression were met, and there was no evidence of multicollinearity among the independent variables. After adjusting for all covariates, social media engagement was inversely associated with perceived mental health, with online engagement linked to poorer perceived mental health (Adjβ = -0.080; p = 0.002). In contrast, email communication was positively associated with perceived mental health, with individuals who sent and received emails reporting better self-rated mental health (Adjβ = 0.113; p < 0.001). Furthermore, no significant relationship was found between perceived mental health and the use of instant messaging apps, online voice or video calls, dating websites, or uploading self-created content. Among sociodemographic factors, individuals aged 65 and older reported better perceived mental health compared to those aged 55–64 (Adjβ = 0.230; p < 0.001). Those who were employed, held a university degree, and had an income exceeding $42,256 reported higher perceived mental health. Moreover, residents of Quebec reported significantly better perceived mental health than those living in Ontario (Adjβ = 0.326; p < 0.001). Households with two residents also reported better perceived mental health compared to single-person households.

**Table 2:** Results of multivariate regression analyses of the association between digital communication, and socio-demographic factors with perceived mental health.

Factor	Perceived Mental Health		
	Adjusted β (SE)	p-value	95% CI
Activities related to Communication			
Email Communication			
Yes	0.113 (0.034)	**<0.001**	0.047, 0.179
No	ref		
Instant Messaging Usage			
Yes	0.032 (0.027)	0.230	-0.020, 0.085
No	ref		
Social Media Engagement			
Yes	-0.080 (0.025)	**0.002**	-0.130, -0.029
No	ref		
Online Voice and Video Calls			
Yes	0.029 (0.024)	0.229	-0.018, 0.077
No	ref		
Use of Dating Platforms			
Yes	-0.080 (0.063)	0.202	-0.203, 0.043
No	ref		
Content Creation and Sharing			
Yes	-0.031 (0.047)	0.514	-0.123, 0.062
No	ref		
Socio-demographic characteristics			
Gender			
Male	ref		
Female	-0.007 (0.022)	0.740	-0.049, 0.035
Age			
55 to 64 years	ref		
65 years and over	0.230 (0.026)	**<0.001**	0.178, 0.282
Employment status			
Yes	0.090 (0.027)	**0.001**	0.037, 0.142
No	ref		
Education			
High school or less	ref		
Some post-secondary	0.038 (0.026)	0.145	-0.013, 0.089
University degree	0.127 (0.030)	**<0.001**	0.069, 0.185
Province			
Atlantic provinces	0.084 (0.030)	0.006	0.025, 0.144
Quebec	0.326 (0.029)	**<0.001**	0.269, 0.383
Ontario	ref		
Manitoba	0.030 (0.050)	0.542	-0.067, 0.127
Saskatchewan	-0.029 (0.042)	0.489	-0.113, 0.054
Alberta	0.003 (0.039)	0.930	-0.073, 0.080
British Columbia	0.032 (0.032)	0.334	-0.033, 0.096
Immigration status			
Landed immigrant	ref		
Non-landed immigrant	0.033 (0.030)	0.276	-0.026, 0.092
Number of persons in the household			
Single-person household	ref		
Two-person household	0.127 (0.025)	**<0.001**	0.077, 0.176
Household of three or more	0.022 (0.039)	0.581	-0.055, 0.098
Income			
<= $42,256	ref		
$42,257 - $72,366	0.149 (0.030)	**<0.001**	0.090, 0.209
$72,367 - $107,480	0.198 (0.035)	**<0.001**	0.129, 0.267
$107.481 - $163,750	0.199 (0.041)	**<0.001**	0.120, 0.279
>$163,750	0.262 (0.042)	**<0.001**	0.180, 0.345

## Discussion

This study aimed to examine the association between digital communication tools usage and self-rated perceived mental health among older adults aged 55 and above, a topic of increasing importance given the rapid growth of digital technology and Canada’s aging population. The results revealed that more than half of the older adults engaged in digital communication activities, such as sending and receiving emails, using instant messaging applications, and accessing social networking websites. The associations between specific digital communication tool use and perceived mental health varied, with some tools linked to more positive and others to more negative perceptions of mental health. The findings indicated that using email as a digital communication tool was associated with better perceived mental health (Adjβ = 0.113; p < 0.001), while engaging in social network use was linked to poorer perceived mental health (Adjβ = -0.080; p = 0.002). These results highlight the association between digital communication tool usage and the mental health of older adults in Canada, providing valuable insights for developing interventions and policies that support the well-being and healthy aging of older adults in an increasingly digital world.

Findings of our study indicated that sending and receiving emails were linked to better perceived mental health. This result was consistent with a previous longitudinal study of older adults aged 50 and above in England, which found that email use for communication was linked to improved mental health [8]. This association may be attributed to the central role of communication in promoting positive mental health, as it enables individuals to maintain strong social connections with family and friends, which in turn alleviates the negative effects of social isolation and loneliness on mental health [2,8]. Additionally, another plausible explanation is that email, as a more structured and asynchronous form of communication, may reduce stress and anxiety compared to more instantaneous methods like instant messaging apps. This may be particularly relevant for older adults, as the ability to read and respond to emails at their own pace can provide them with a greater sense of control over interactions [23].

Furthermore, results revealed that social media engagement among older adults is associated with poorer perceived mental health outcomes, consistent with previous literature that highlights the potential negative effects of social media use on mental well-being [17,24]. While existing studies on this topic presents mixed findings, these variations may stem from differences in study designs, definitions of social media engagement, measurement of outcome variables, and the inclusion of various covariates. One possible explanation for the negative association is that social media may expose older adults to distressing content and promote social comparison, both of which could adversely affect their mental health [17]. For instance, studies of adults aged 60 and older in the United States found that certain online social activities were linked to poorer mental health outcomes, but this association varied based on the type of online engagement. For instance, individuals who reached out to check on someone missing from an online community reported increased feelings of depression, while those participating in online discussions about health and aging reported greater anxiety [17,24]. Additionally, social media use during the COVID-19 pandemic, driven by isolation and loneliness, may have further exacerbated mental health issues among older adults. A study of adults aged 65 and older in California suggested that social media engagement during the pandemic, influenced by feelings of isolation and loneliness, was associated with exposure to distressing content, may be linked to poorer mental health outcomes [25].

The findings showed no significant associations between instant messaging and mental health in older adults. While instant messaging facilitates rapid communication, it may lack the depth and richness needed for meaningful social engagement, making face-to-face interactions more impactful for emotional well-being and reducing loneliness [26]. Similarly, no significant association was found between video calls use and mental health outcomes. This contrasts with a previous study conducted in the United States, which reported that older adults who used video chat technologies such as Facetime and Skype had a significantly lower risk of developing depressive symptoms, likely because these platforms mimic in-person social interaction and enhances connectedness [27]. However, these differing findings could be due to variations in study populations, research design and methods, or the frequency and context of video call usage among older adults. Additionally, the use of dating websites and content-sharing platforms was not linked to mental health outcomes in this study. This may be due to the lower usage rates among older adults, which could have limited the statistical power to detect potential associations rather than indicating a lack of relevance to mental health.

Regarding sociodemographic factors, individuals aged 65 and above reported better perceived mental health than those aged 55–64. This may be due to older adults experiencing less work-related anxiety and stress after retirement, thereby enhanced mental well-being [28]. Additionally, retired older adults often have greater autonomy and more time to build social networks and increase their social capital, which can positively influence their mental health [29]. Moreover, employment was associated with better perceived mental health in older adults. This aligns with global studies showing that employment enhances mental health by providing a sense of purpose, promoting social interaction, and reducing feelings of isolation and loneliness [30–32]. For example, a longitudinal study of seniors in Singapore found that working older adults had better cognitive function, fewer depressive symptoms, and improved mental health [31]. This association might also be attributed to a smoother transition from employment to retirement, where material resources and socio-economic status play a critical role [33].

Older adults with higher income also reported improved mental health. A higher income level provides the opportunity for older adults to attend more social functions, enhances social engagements, and thereby improves mental well-being. The availability of financial resources can also support self-esteem, promoting more social connections and reducing feelings of loneliness, and ultimately contributing to better mental well-being [34]. Similarly, higher educational attainment was positively linked to better perceived mental health outcomes. This aligns with previous study that found a positive relationship between higher education levels and psychological well-being [35]. Education is recognized as a key factor in enabling social engagements and promoting the overall well-being of older adults, potentially contributing to a more positive outlook on life and improved mental health [36].

The results also showed that living arrangement of older adults is linked to mental health outcomes. Individuals living in two-person household reported significantly better perceived mental health than those living alone. This association may stem from the increased social interaction and emotional support that cohabitation provides, which helps reduce loneliness and isolation [37,38]. Additionally, shared responsibilities and financial burdens may alleviate stress, contributing to overall well-being. Lastly, the residents of Quebec reported better perceived mental health compared to those in Ontario. This finding aligns with the 2024 State of Mental Health in Canada report, which noted that the prevalence of negative mental health outcomes, such as mood disorders, anxiety, and depression, was lower among individuals in Quebec than in Ontario [39]. This difference may be partially attributed to Quebec’s lower cost of living and greater housing affordability, factors that can positively impact the mental well-being of older adults, particularly in contrast to Ontario [39,40]. Additionally, Quebec’s stronger emphasis on community-based social services, broader social protection policies, and more integrated primary and mental healthcare delivery may contribute to greater social support and lower psychosocial stress, influencing perceived mental health [41,42].

### Limitations

This study is one of the first to examine the association between various digital communication tools and self-rated mental health in the general older adult population in Canada. Nonetheless, several limitations should be considered. First, due to the cross-sectional design of the study, causal relationships cannot be established, and reverse causation is possible whereby individuals with better mental health may be more likely to engage in certain digital communication activities. Second, there is a potential for information bias, as all responses were self-reported, and the primary dependent variable measured perceived overall mental health rather than using a validated and objective assessment. Third, the generalizability of the results may be limited due to the exclusion of individuals residing in long-term institutions. In addition, the findings are restricted to older adults who reported Internet use in the past three months and may not generalize to those with no recent Internet use. Fourth, digital communication tool use in the CIUS 2022 survey is measured as a binary indicator and does not capture frequency, duration, or context of use, nor distinguish between active and passive engagement. This dichotomization may result in limited depth of information, potential misclassification, and limits the ability to assess dose–response relationships. Fifth, the results may be affected by residual confounding, as data were not available on several important factors that may influence both digital communication use and mental health outcomes, including pre-existing mental health conditions, chronic illness, functional limitations, and rural or urban residence.

## Conclusion

The findings of this study offer valuable insights into the complex relationship between digital communication tool usage and mental health of older adults in Canada. Results indicated that email communication was associated with better mental health, while social media use was linked to poorer outcomes. These findings underscore the need for targeted public health interventions to support the mental well-being of older adults in today’s digital landscape. Initiatives such as educational campaigns on responsible social media use, assessing the credibility of online information, and recognizing potential risks may help empower older adults to engage more effectively and safeguard their mental health. However, further research is needed to evaluate the effectiv‌‌eness of these approaches in improving mental health outcomes among older adults.

## Supporting information

S1 Appendix Survey Questions. (DOCX)
