# Supplementary material for: The association between digital communication tool use and perceived mental health among older adults in Canada
Source: PLOS Glob Public Health. 2026 Jun 24;6(6):e0006540. doi: 10.1371/journal.pgph.0006540 (PMC13293427; doi:10.1371/journal.pgph.0006540)
Supplement: S1 Appendix — (DOCX) [file pgph.0006540.s001.docx]

**S1 Appendix**

| Variable | Code | Explanation and/or question | Response options |
| --- | --- | --- | --- |
| General Health | GH_R01 | The following questions are about health. By health, we mean not only the absence of disease or injury but also physical, mental and social well-being. |  |
| General Health | GH_Q010A | In general, how is your health? | 1: Excellent  2: Very good  3: Good  4: Fair  5: Poor |
| Activities related to Communication |  | During the past three months, which of the following activities, related to communication, have you done over the Internet? |  |
| Email Communication | UI_Q010A | Have you: Sent and received emails | 1:Yes  2:No |
| Instant Messaging Usage | UI_Q010B | Have you: Sent messages using an instant messaging app, e.g., iMessage, Facebook Messenger, WhatApp, Signal | 1:Yes  2:No |
| Social Media Engagement | UI_Q010C | Have you: Used social networking websites or apps, e.g., Facebook, Twitter, LinkedIn, Instagram | 1:Yes  2:No |
| Online Voice and Video Calls | UI_Q010D | Have you: Made online voice calls or video calls, e.g., Zoom, Google Meet, Facetime, VoIP | 1:Yes  2:No |
| Use of Dating Platforms | UI_Q010E | Have you: Used dating websites or apps | 1:Yes  2:No |
| Content Creation and Sharing | UI_Q010F | Have you: Uploaded self-created content on sharing websites, a blog or a personal website, e.g., YouTube, Flickr  Exclude social networking websites or apps. | 1:Yes  2:No |
| Socio-demographic characteristics |  |  |  |
| Gender | GDR_Q010 | What is your gender?  Is it: | 1: Male  2: Female  3: Please specify |
| Age | AGE_Q01 | Age Groups - Derived variable  What is your date of birth? | YYYY/MM/DD |
| Employment status | EMP | Information derived using LMAM_Q01, LMAM_Q02 and LMAM_Q03. |  |
|  | LMAM_Q01 | Last week, did you work at a job or business? | 1: Yes  2: No |
|  | LMAM_Q02 | Last week, did you have a job or business from which you were absent? | 1: Yes  2: No |
|  | LMAM_Q03 | What was the main reason you were absent from work last week? | 01: Vacation  02: Own illness or disability  03: Caring for own children  04: Caring for elder relative (60 years of age or older)  05: Maternity or parental leave  06: Other personal or family responsibilities  07: Labour dispute (strike or lockout) (Employees only)  08: Temporary layoff due to business conditions (Employees only)  09: Seasonal layoff (Employees only)  10: Casual job, no work available (Employees only)  11: Work schedule (e.g., 10 days on, 10 days off, employees only)  12: Self-employed, no work available (Self-employed only)  13: Seasonal business (Excluding employees)  14: Other |
| Education | ED_Q05 | What is the highest certificate, diploma or degree that you completed? | 1: Less than high school diploma or its equivalent  2: High school diploma or a high school equivalency certificate  3: Trades certificate or diploma  4: College, CEGEP or other non-university certificate or diploma (other than trades certificates or diplomas)  5: University certificate or diploma below the bachelor's level  6: Bachelor's degree (e.g., B.A., B.A. (Hons), B.Sc., B.Ed., LL.B.)  7: University certificate, diploma or degree above the bachelor's level |
| Province | Province | Information derived using postal codes. |  |
| Immigration status | IMM_STA | Landed immigrants are permanent residents who have indicated a year of landing in  Canada since 1952. Variable derived from LANDING_YEAR, IMDB.  Source: Longitudinal Immigration Database (IMDB) |  |
| Number of persons in the household | RRS_G12 | Including yourself, how many persons are staying in your household? | 1: One person household  2: Two person household  3: Three person household  4: Four person household  5: Five or more person household |
| Income | HINCQUIN | Census family income quintile - Derived variable  Source: Annual Income Estimates for Census Families and Individuals (T1 Family File) |  |
